# Human Amygdala Volumetric Patterns Convergently Evolved in Cooperatively Breeding and Domesticated Species

**DOI:** 10.1007/s12110-023-09461-3

**Published:** 2023-09-22

**Authors:** Paola Cerrito, Judith M. Burkart

**Affiliations:** 1 Collegium Helveticum, ETH Zürich, Schmelzbergstrasse 25, Zürich, 8092 Switzerland; 2 Department of Evolutionary Anthropology, University of Zurich, Zürich, Switzerland; 3 Center for the Interdisciplinary Study of Language Evolution (ISLE), University of Zurich, Zürich, Switzerland

**Keywords:** Amygdala, Cooperative breeding, Human self-domestication, Reactive aggression, Evolutionary cognition

## Abstract

The amygdala is a hub in brain networks that supports social life and fear processing. Compared with other apes, humans have a relatively larger lateral nucleus of the amygdala, which is consistent with both the self-domestication and the cooperative breeding hypotheses of human evolution. Here, we take a comparative approach to the evolutionary origin of the relatively larger lateral amygdala nucleus in humans. We carry out phylogenetic analysis on a sample of 17 mammalian species for which we acquired single amygdala nuclei volumetric data. Our results indicate that there has been convergent evolution toward larger lateral amygdala nuclei in both domesticated and cooperatively breeding mammals. These results suggest that changes in processing fearful stimuli to reduce fear-induced aggression, which are necessary for domesticated and cooperatively breeding species alike, tap into the same neurobiological proximate mechanism. However, humans show changes not only in processing fearful stimuli but also in proactive prosociality. Since cooperative breeding, but not domestication, is also associated with increased proactive prosociality, a prominent role of the former during human evolution is more parsimonious, whereas self-domestication may have been involved as an additional stepping stone.

**Supplementary Information:**

The online version contains supplementary material available at 10.1007/s12110-023-09461-3.

The amygdala has been described as a hub in brain networks that supports social life (Bickart et al., 2014; Wu & Hong, 2022). In humans, compared with other apes, the lateral nucleus of the amygdala is relatively larger (Barger et al., 2007) and contains more neurons (Barger et al., 2012). Recent work has demonstrated that, in humans, reduced amygdala volume correlates with a reduction in the ability to both feel fear and recognize fear in others, and with an increased prevalence of psychopathic traits (Marsh et al., 2008). Similarly, extreme altruists are characterized by a heightened ability to recognize fear, and by a larger and more active amygdala (Marsh et al., 2014). These neuroanatomical findings appear to be well in line with the self-domestication (SD) hypothesis of human evolution (Hare, 2017), which argues for selection against fear-induced aggression toward strangers given that the amygdala and particularly the lateral nucleus play a critical role in fear processing.

However, this relative increase in lateral amygdala nucleus volume in humans compared with other apes is also consistent with the cooperative breeding model (CB) of human evolution. The CB model (Hrdy & Burkart, 2020) posits that infant care by non-mothers (allomaternal care) became essential for infant survival during the Pleistocene. Indeed, our life history pattern, which includes short interbirth intervals without compromising infant size and/or brain size, depends, energetically, on the availability of helpers in raising offspring (Cerrito & Spear, 2022). Importantly, this reliance on allomaternal care requires that immatures overcome the fear of being close to conspecifics who are not their mothers, even if those conspecifics are in the possession of valuable food sources in the case of provisioning. Moreover, mothers need to overcome the fear of others approaching, handling, and carrying their infants. These two different types of fear and consequent aggressive responses (affective leading to defensive) make use of the same underlying brain networks, as verified across several species of mammals (Panksepp, 2004).

In sum, both SD and CB require overcoming fear, tolerating close proximity, and eventually eliciting care from others: in domesticated species from humans, and in cooperatively breeding ones from conspecifics. Both thus had to find ways to deal with these potentially fear-eliciting situations. We therefore predict similar neuroanatomical adaptations of the amygdala nuclei in both domesticated and cooperatively breeding species. We take a comparative approach to test whether the amygdala volumetric patterns observed in humans are more likely the result of SD or CB. Specifically, we hypothesize that a relatively larger lateral nucleus, which is the sensory interface in fear conditioning (LeDoux et al., 1990), allowed for the evolution of a heightened sensitivity in the recognition of others’ fear, expressed also, especially in cooperatively breeding species, via facial expressions (Cerrito & DeCasien, 2021). In humans, this increased ability to recognize others’ fear is thought to trigger a violence inhibition mechanism (Blair, 1995) which in turn allows for adults to care for infants that are not their own (Marsh, 2019).

To test our hypothesis, we combine neuroanatomical data with detailed data on infant care and domestication status and use phylogenetic methods to assess the correlation between relative volumes of different amygdala nuclei, domestication, and CB. Specifically, we test whether convergent evolution resulted in similar neuroanatomical adaptations in the amygdala of domesticated and cooperatively breeding species, with both groups having a larger than expected lateral nucleus than species who are neither domesticated nor cooperative breeders.

## Materials and Methods

We compiled previously published volumetric data from 64 adult individuals of 17 mammalian species (of which three are domesticated and three are cooperative breeders) for the following regions: the amygdaloid complex (AC) and the basolateral nucleus (BLA) and its lateral (L), basal (B), and accessory basal (AB) nuclei (the latter three comprise the BLA). The species analyzed in the study are *Sorex araneus, Pongo pygmaeus, Oryctolagus cuniculus, Cavia porcellus, Sus scrofa, Macaca nemestrina, Macaca fascicularis, Pan paniscus, Gorilla gorilla, Rattus norvegicus, Macaca mulatta, Pan troglodytes, Hylobates lar, Sapajus apella, Vulpes vulpes, Callithrix jacchus*, and *Homo sapiens.* In the dataset provided in the ESM for each individual of each species we report the sample size (*n*), sex, and age (in years). All data come from adult individuals, with a balanced representation of each sex. All volumetric data were obtained from the literature (Barger et al., 2007; Carlo et al., 2010; Chareyron et al., 2011, 2012; Równiak et al., 2022). When both left and right hemispheric values are reported, the mean was taken. Conversely, in some cases (Chareyron et al., 2011, 2012), the right or left hemisphere was randomly chosen after ascertaining the absence of lateralization (*p* = 0.757), and in yet others (Carlo et al., 2010) only the left is reported. The variance of volumetric measures is quite small within a species (Chareyron et al., 2011; Równiak et al., 2022); hence, data for species with even small samples sizes is expected to be representative of the taxon. All behavioral data were taken from published literature (Cerrito & Spear, 2022; Isler & van Schaik, 2012). For a definition of the measures of allomaternal care, see Isler and van Schaik (2012).

We created a binary variable aimed at expressing whether a species was a cooperative breeder. To do so we first created a compound “allocare_sum” variable as the sum of all forms of allomaternal care observed in each species: male provisioning of infants, provisioning of infants by other group members, carrying of infants by males, carrying of infants by others, allonursing, protection by male, and other forms of infant care, such as thermoregulation, babysitting, and pup retrieval (called communal work). We then made this continuous variable (which is the sum of all the single allocare variables) binary (non–cooperative breeder = 0 to 2.64; cooperative breeder = 2.64 to 5.2) using *k*-means clustering implemented using the “discretize” function in the R package *arules* (Hornik et al., 2005). Finally, we made a compound binary variable, called “elicit_care,” which takes the value of 1 for species that are either domesticated or cooperative breeders, or the value of 0 for those that are neither. Given that in some species there is no infant carrying at all (neither maternal nor allomaternal), we also created an “allocare_sum_NoCarry” variable which represents the sum of all allomaternal care behaviors except infant carrying. The breaks used to discretize this variable are: non–cooperative breeder = 0 to 2.36; cooperative breeder = 2.36 to 4.5. The resulting species identified as cooperative breeders are the same three as in the “allocare_sum” variable, and therefore the value of elicit_care for each species does not change whether one selects the “allocare_sum” or the “allocare_sum_NoCarry” value. The final and complete dataset is provided in the ESM, where volume is expressed in cubic centimeters (cm^3^).

We carried out all statistical analyses using R version 4.0.1 (R Core Team, 2020). We used phylogenetic generalized least squares (PGLS) regressions to assess the correlation between the variable “elicit_care” (domestication or cooperative breeding) and relative neuroanatomical data. To compare the data across species with a large range of variation in amygdala size, we regressed B, L, and AB against BLA and against the entire AC. We used the additive effect of “elicit_care” to test for significant differences between intercepts. We assessed the correlation between infant care measures on one side and relative neuroanatomical data on the other using phylogenetic generalized least squares (PGLS) regressions (Grafen, 1989). The models used a recently updated species-level mammals phylogeny (Upham et al., 2019). We used the maximum clade credibility tree calibrated using node dates and an exponential prior. To account for the high degree of skew, we log-transformed (in base *e*) all the volumetric, weight, and time variables.

We estimated λ for the PGLS models independently for each regression using maximum likelihood. We carried out the PGLS analyses using the “pgls” function in the R package *caper* (Orme et al., 2013). For each significant model we also report the effect size, expressed as percentage. To compare the effect size between different explanatory variables, we scaled the intercept difference to reflect the maximum value that the variable can take, so the intercept difference is multiplied by that value and then the difference in the natural log of the effect sizes is exponentiated to convert back to the original ratio of the effect sizes. We then proceeded to compute the AIC (Akaike, 1998) for each of the significant models and compared models with the same response variable. The complete list of models, their estimates, and the results of the model selection using AIC are available in Table 1. The model with the lowest AIC score is considered the best model, and models with an AIC score within 2 of that of the best model are also considered to receive support (Akaike, 1998).

**Table 1:** Estimates of the models in which each nucleus is regressed against the basolateral one or the entire amygdaloid complex (AC), plus the additive term tested. For each model: response variable, explanatory variable plus additive term, *p*-value, R^2^, intercept difference, adjusted intercept difference (see methods), and effect size (in %). **Bold** values are significant. “Best” indicates that the model has the lowest AIC score for that response variable and no other model is within 2; “Tied” indicates that the model either has the lowest AIC but there are others within 2, or that its AIC is within 2 of the lowest; “No” indicates that the AIC score is > 2 from the lowest AIC score for that response variable

Response	Predicted by	*p*	R^2^	Intercept difference	Adjusted intercept	Effect size	AIC
**Lateral**	**Basolateral + Elicit_binary**	**0.000**	**0.996**	**0.353**	**0.353**	**42.316**	**Best**
**Lateral**	**Basolateral + Cooperative_breeder**	**0.014**	**0.987**	**0.304**	**0.304**	**35.592**	**No**
**Lateral**	**Basolateral + Domesticated**	**0.037**	**0.993**	**0.302**	**0.302**	**35.190**	**No**
Accessory_Basal	Basolateral + Cooperative_breeder	0.565	0.996	−0.054		0.000	
Accessory_Basal	Basolateral + Domesticated	0.713	0.996	0.035		0.000	
Accessory_Basal	Basolateral + Elicit_binary	0.863	0.996	−0.013		0.000	
**Basal**	**Basolateral + Cooperative_breeder**	**0.001**	**0.990**	**−0.281**	**−0.281**	**−24.520**	**No**
**Basal**	**Basolateral + Elicit_binary**	**0.000**	**0.995**	**−0.298**	**−0.298**	**−25.790**	**Best**
**Basal**	**Basolateral + Domesticated**	**0.012**	**0.995**	**−0.307**	**−0.307**	**−26.471**	**No**
**Lateral**	**AC + Elicit_binary**	**0.000**	**0.992**	**0.473**	**0.473**	**60.504**	**Best**
**Lateral**	**AC + Cooperative_breeder**	**0.004**	**0.978**	**0.381**	**0.381**	**46.379**	**No**
**Lateral**	**AC + Domesticated**	**0.040**	**0.988**	**0.380**	**0.380**	**46.286**	**No**
Accessory_Basal	AC + Cooperative_breeder	0.699	0.993	0.048		0.000	
Accessory_Basal	AC + Domesticated	0.373	0.993	0.114		0.000	
Accessory_Basal	AC + Elicit_binary	0.315	0.993	0.099		0.000	
**Basal**	**AC + Elicit_binary**	**0.002**	**0.981**	**−0.290**	**−0.290**	**−25.172**	**Best**
**Basal**	**AC + Cooperative_breeder**	**0.003**	**0.981**	**−0.290**	**−0.290**	**−25.210**	**No**
Basal	AC + Domesticated	0.465	0.983	−0.137		0.000	

Since the results were significant, we proceeded to conduct post-hoc tests to assess which of the constituting variables of “elicit_care” (i.e., domestication and cooperative breeding) were, individually, correlated with significant nuclei volumetric differences.

For each continuous variable we measured the phylogenetic signal using Blomberg’s K (Blomberg et al., 2003) implemented by the “phylosig” function in the R package *phytools* (Revell, 2012). To test significance, we used 100,000 iterations. For discrete variables we followed previously published methods (Cerrito & Spear, 2022) and estimated the phylogenetic signal using δ (Borges et al., 2019), and ran 10,000 iterations to test for significance. The value of K/δ for each of the variables analyzed is available in Table 2.

Finally, we used the Wheatsheaf Index (Arbuckle et al., 2014) to test for convergent evolution of nuclei volumes in cooperatively breeding and domesticated species. This index allows us to explicitly test hypotheses regarding the evolutionary convergence of quantitative traits by incorporating both phenotypic similarities and phylogenetic relatedness. We implemented this using the “test.windex” function in the R package *windex* (Arbuckle & Minter, 2015), which performs a statistical test for convergence. We defined as focal species the ones that are either cooperative breeders or domesticated ones (as per data in the ESM) and used 10,000 iterations to test for significance.

**Table 2:** Estimated phylogenetic signal for all the variables used in the analyses. Blomberg’s K was used for continuous variables, while ẟ was used for discrete ones. Phylogenetic signal is significant for all neuroanatomical variables, and it is very strong (K > 1). Phylogenetic signal is not significant for domestication or cooperative breeding

Variable	K/*𝛿	*p*	Simulations
Amygdaloid complex	1.4559	0.000020	100000
Basolateral nucleus	1.6061	0.000030	100000
Lateral nucleus	1.2900	0.000060	100000
Basal nucleus	1.8526	0.000010	100000
Accessory basal nucleus	1.5721	0.000020	100000
Elicit binary	*1.0749	0.430000	10000
Cooperative breeder	0.8708	0.633000	10000
Domesticated	*25.83	0.203400	10000

## Results

**Fig. 1:**
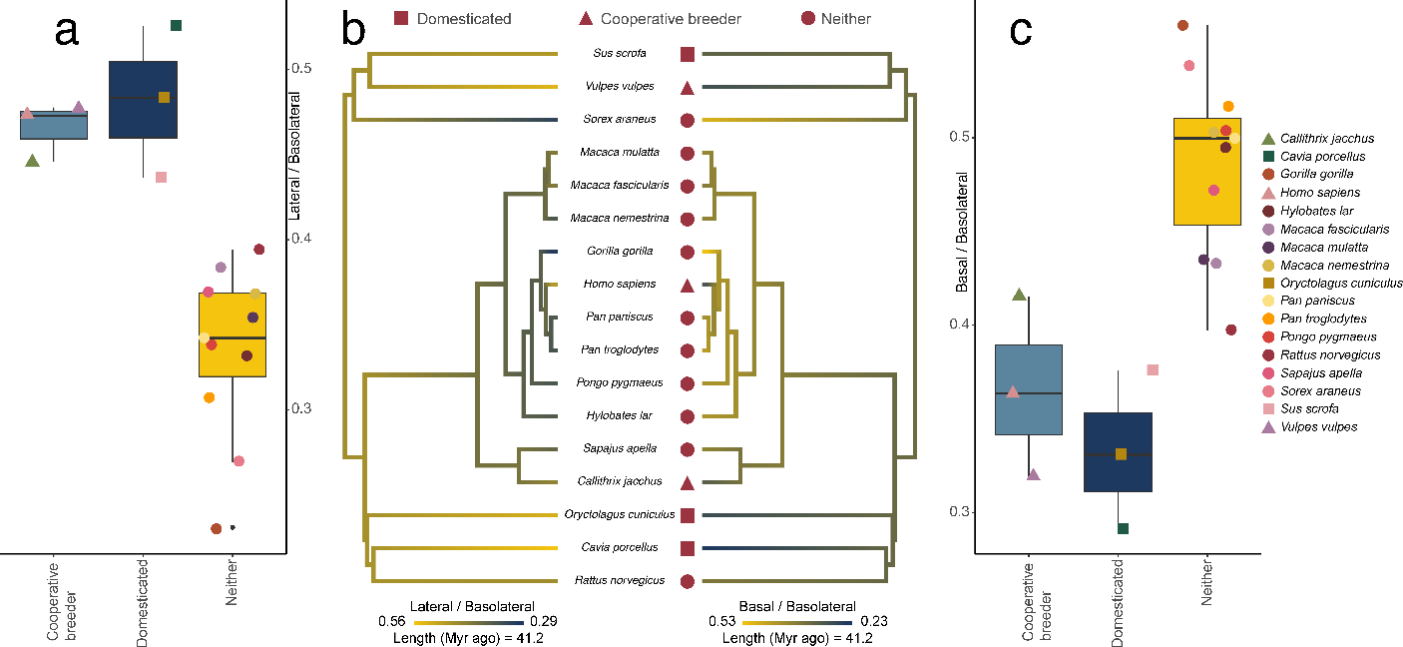
(a) Boxplot indicating the ratio between lateral and basolateral nuclei volumes in cooperative breeders, domesticated species, and others. Each dot represents a species, the legend for the different colors is the same one as reported in panel C. Pairwise corrected p-values of phylogenetic ANOVAs are: 0.003 for *Cooperative breeders* and *Neither*, and 0.018 for *Domesticated* and *neither*. (b) Ancestral state reconstruction of lateral to basolateral volume (left) and basal to basolateral volume (right). In both domesticated species and cooperative breeders, the lateral nucleus is relatively larger (yellow) and the basal one is relatively smaller (blue). (c) Boxplot indicating the ratio between basal and basolateral nuclei volumes in cooperative breeders, domesticated species, and others. Each dot represents a species, identified by a color. Pairwise corrected p-values of phylogenetic ANOVAs are: 0.003 for *Cooperative breeders* and *Neither*, and 0.03 for *Domesticated* and *neither*. Branch lengths indicate time (in millions of years ago). The figure was created using R statistical software (R Core Team, 2020) v. 4.2.2, and the packages *phytools* (Revell, 2012) and *ggplot2* (Wickham & Chang, 2016)

Our findings suggest that both domesticated and cooperatively breeding species share adaptations in the amygdala that are fundamentally involved in processing fear-eliciting stimuli (Manassero et al., 2018; Ostroff et al., 2010). First, both cooperative breeding and domestication are associated with a significant increase of the volume of the lateral nucleus relative to BLA (+ 35% in both cases) and a relative decrease in the basal one (− 24% and − 26%, respectively; Fig. 1). These results are robust and very similar when regressing L and B against AC: a 46% relative increase in L for both groups, a 25% relative decrease in B for cooperatively breeding species, but no significant effect for domesticated ones. Further, we find that the model with the compound variable “elicit_care” as explanatory variable is the best one (according to the AIC value) in explaining both the increase of the lateral nucleus and the decrease of the basal one. No significant effect is found for the accessory basal nucleus.

The complete list of all the models tested, their estimates, and results are available in Table 1; the phylogenetic signal for each of the variables used is reported in Table 2.

**Fig. 2:**
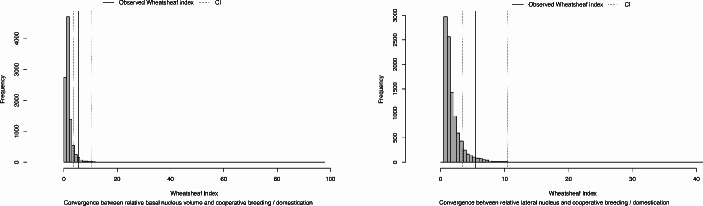
Left: Wheatsheaf Index of the convergence of relative basal nucleus volume in cooperatively breeding and domesticated species (W = 5.49; 95% CI = 3.65, 10.31; *p* = 0.03). Right: Wheatsheaf Index of the convergence of relative lateral nucleus volume in cooperatively breeding and domesticated species (W = 5.38; 95% CI = 3.42, 10.49; *p* = 0.04). The figure was created using R statistical software (R Core Team, 2020) v. 4.2.2, and the package *windex* (Arbuckle & Minter, 2015)

Second, to test for convergence, we calculate the Wheatsheaf Index (W). The 10,000 iterations (Fig. 2) support the hypothesis of convergent evolution in cooperative breeding and domesticated species: (i) relative (to BLA) lateral nucleus volume (W = 5.38; *p* = 0.04; 95% CI = 3.42, 10.49; *p* = 0.04); (ii) relative (to BLA) basal nucleus volume (W = 5.49; *p* = 0.03; 95% CI = 3.65, 10.31; *p* = 0.03). We thus find robust evidence for a relatively larger lateral nucleus in cooperatively breeding and domesticated species that is the result of convergent evolution.

## Discussion

Our results suggest that reduced reactive aggressiveness, mediated by a relatively larger lateral nucleus, constitutes a shared adaptation in cooperatively breeding and domesticated species. Both processes, domestication and cooperative breeding, apparently tap into the same proximate mechanism: a neurobiological adaptation of the lateral amygdala, likely relating to fear processing and reactive aggressiveness, which is adaptive in both cases. For domesticated animals, a reduction of fear-induced aggressiveness toward humans provides adaptive advantages (e.g., in terms of food sharing for commensal domestication), as in the case of dogs. Such a relationship is asymmetrical, and the roles do not change over time. Conversely, in cooperatively breeding species the selective pressure for increased social tolerance is entirely different as it is directed toward conspecifics, it confers the adaptive advantage of socially distributing the energetic cost of raising offspring, and it is susceptible to change over time with helpers becoming breeders themselves such that a given individual can take up different roles in the cooperative relationship as it matures.

Hence, what could likely be selected for in both CB and domesticated species is the reactive response to fear.

Further inquiries, with increased samples sizes, regarding the role played by the basal nucleus are warranted by our preliminary results showing that this nucleus (relative to total amygdala volume) is reduced only in cooperatively breeding species and not in domesticated ones. The basal nucleus is implicated, together with the nucleus accumbens, in the avoidance response (Ramirez et al., 2015). A relatively smaller nucleus could potentially correlate with a higher threshold that must be met by a stimulus before eliciting avoidance, thus favoring an approach response. This could be instrumental for mothers to allow others to care for their offspring, and for offspring, that individuals other than their mothers may care for them.

What are the implications of these findings for human evolution? The human social niche not only required changes in the processing of fearful stimuli and a reduction in fear-induced aggression, but also an increase in proactive prosociality—in other words, an other-regarding concern for the welfare of others (Burkart et al., 2007). Whereas reduced fear-induced aggression can result in increased social tolerance, proactive prosociality goes beyond that by actively motivating behaviors that benefit others. A reduction in reactive aggressiveness, potentially mediated by the derived amygdala volumetric patterns found in the present research, is thus only a prerequisite or stepping stone for the emergence of proactive prosociality. Whereas most domesticated species are not particularly prosocial, with dogs even losing this feature during the domestication process (Range & Marshall-Pescini, 2022), empirical evidence across a large number of species shows that cooperative breeding is associated with proactive prosociality (Burkart et al., 2014; Horn et al., 2020). Furthermore, recent research shows that our less reactively aggressive close relatives who arguably underwent self-domestication, the bonobos, nonetheless show low levels of prosociality when tested with the same paradigms (Verspeek et al., 2022). Our results thus indicate that the presence of reduced reactive aggressiveness in humans may be equally linked to self-domestication or cooperative breeding, but proactive prosociality requires additional selection pressures that are most likely related to cooperative breeding. Our results thus contribute to disentangling the roles of self-domestication and cooperative breeding during human evolution (Range & Marshall-Pescini, 2022; Sánchez-Villagra & van Schaik, 2019).

### Electronic Supplementary Material

Below is the link to the electronic supplementary material.

Supplementary Material 1

## Data Availability

All data generated or analyzed during this study are included in this published article and the attached ESM.
